# Spinel cobalt-based binary metal oxides as emerging materials for energy harvesting devices: synthesis, characterization and synchrotron radiation-enabled investigation†

**DOI:** 10.1039/d4ra03462g

**Published:** 2024-07-04

**Authors:** Abdelelah Alshanableh, Yusuf Selim Ocak, Bashar Aljawrneh, Borhan Aldeen Albiss, Khaled Shawakfehc, Latif U. Khane, Messaoud Harfouchee, Saja Alrousan

**Affiliations:** a Nanotechnology Institute, Jordan University of Science & Technology PO Box 3030 Irbid 22110 Jordan; b Department of Physics and Engineering Physics, Morgan State University Baltimore Maryland 21234 USA; c Department of Physics, Al-Zaytoonah University of Jordan PO Box 130 Amman 11733 Jordan B.Aljawarneh@zuj.edu.jo; d Department of Chemistry, Jordan University of Science & Technology PO Box 3030 Irbid 22110 Jordan; e Synchrotron-Light for Experimental Science and Applications in the Middle East (SESAME) PO Box 7 Allan 19252 Jordan

## Abstract

The synthesis and characterization of spinel cobalt-based metal oxides (MCo_2_O_4_) with varying 3d-transition metal ions (Ni, Fe, Cu, and Zn) were explored using a hydrothermal process (140 °C for two hours) to be used as alternative counter electrodes for Pt-free dye-sensitized solar cells (DSSCs). Scanning electron microscopy (SEM) and atomic force microscopy (AFM) revealed distinct morphologies for each metal oxide, such as NiCo_2_O_4_ nanosheets, Cu Co_2_O_4_ nanoleaves, Fe Co_2_O_4_ diamond-like, and Zn Co_2_O_4_ hexagonal-like structures. The X-ray diffraction analysis confirmed the cubic spinel structure for the prepared MCo_2_O_4_ films. The functional groups of MCo_2_O_4_ materials were recognized in metal oxides throughout Fourier transform infrared (FTIR) analysis. The local structure analysis using X-ray absorption fine structure (XAFS) at Fe and Co K-edge identified octahedral (Oh) Co^3+^ and tetrahedral (Td) Co^2+^ coordination, with Zn^2+^ and Cu^2+^ favoring Td sites, while Ni^3+^ and Fe^3+^ preferred Oh active sites. Further investigations utilizing the Fourier transformation (FT) analysis showed comparable coordination numbers and interatomic distances ranked as Co–Cu > Co–Fe > Zn–Co > Co–Ni. Furthermore, the utilization of MCo_2_O_4_ thin films as counter electrodes in DSSC fabrication showed promising results. Notably, solar cells based on CuCo_2_O_4_ and ZnCo_2_O_4_ counter electrodes showed 1.9% and 1.13% power conversion efficiency, respectively. These findings indicate the potential of employing these binary metal oxides for efficient and cost-effective photovoltaic device production.

## Abstract 2

The synthesis and synchrotron-based characterization of MCo_2_O_4_ with 3d-transition metal ions (Ni, Fe, Cu, Zn) were explored using a hydrothermal process, revealing distinct morphologies and promising potential as counter electrodes for DSSCs.

## Graphical abstract

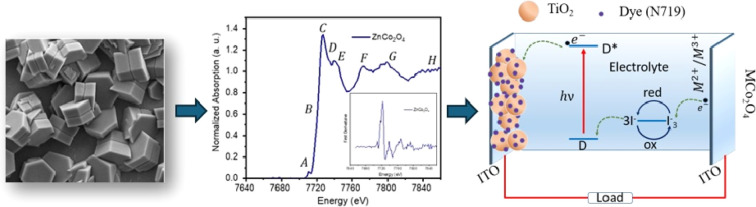

## Introduction

Because of the permanent growth in the global population and industrialization, the demand for energy has increased steadily. Limits in fuel-based energy sources make the need for cleaner, sustainable alternative energy sources inevitable. Among all renewable energy sources, solar energy is one of the most impressive ones. Photovoltaic technologies, particularly dye-sensitized solar cells (DSSCs), have emerged as key factors in this transition owing to their low cost and easy production processes. Another important parameter that increases the impression of DSSCs is the capability of harvesting energy from even lower-intensive light.

Significant effort has been devoted to increasing the power conversion efficiency and stability of DSSCs while making them cost-effective. To achieve optimal performance in the DSSCs, the counter electrode commonly incorporates platinum (Pt) on a surface of transparent conductive oxide.^1,2^ Several novel approaches have been formulated to improve the DSSCs' performance including enhancing the light absorption capacity of the sensitizer, charge separation of photo-induced charge at the electrolyte–semiconductor interface, and prompt the migration of charge towards the counter electrode.^3–5^ The proposed approaches involve the binding of a sensitizer onto wide bandgap semiconductor substrates such as titanium dioxide, which aims to enhance light absorption capabilities.^6^ Additional efforts have been made to replace the Pt counter electrode in DSSCs. As an alternative to Pt, various kinds of materials, including metal oxides, metal sulfides, carbon, and hybrid electrodes, have been proposed to obtain cost-effective device fabrication.^7–11^ An alternative candidate with significant potential for DSSCs is spinel cobalt-based metal oxides (MB_2_O_4_, M: tetrahedral, B: octahedral sites). Such unique characteristics involve excellent electrical conductivity, efficient charge transfer, and significantly large surface area. Besides that, various oxidation states, and notable electrochemical activity make them highly advantageous to be used in energy and photocatalytic applications.^12–15^

The spinel cobalt oxide (Co_3_O_4_) is one of the most investigated electro-catalyst materials that is widely applied in the counter electrode for DSSCs. The tetrahedral site (Td) is occupied by Co^2+^ ions, while the octahedral site (Oh) is occupied by two Co^3+^ ions.^16^ To date, Co^2+^ and Co^3+^ serve as active sites within the spinel cobalt metal oxide Co_3_O_4_, and they have been reported to have a crucial role in charge transfer and their catalytic activity.^17–19^ Recent studies report that replacing the 3rd transition metal ions, such as Ni and Zn ions, with the Co^2+^ in spinel cobalt oxide (Co_3_O_4_) enhances their electrical conductivity.^20^ For instance, ZnCo_2_O_4_ and NiCo_2_O_4_ exhibited superior electrical conductivity compared to Co_3_O_4_. Zn^2+^ ions are beneficial for indicating defects in the Td sites.,^17–20^ as observed in spinel oxides, which in turn enhances the catalytic activity for the oxygen evolution reaction. Moreover, the large surface and electrical conductivity of NiCo_2_O_4_ result in better catalytic activity than spinel metal oxide (Co_3_O_4_). Also, the Fe^3+^ in FeCo_2_O_4_ occupies the Oh sites, leading to a shift in energy level near the Fermi level, which improves the activity of spinel metal oxide.^21,22^

Several reports have integrated the spinel-based cobalt structure into DSSC devices. Interestingly, the Co_3_O_4_ counter electrode of PCE ∼8.6% in DSSCs exhibits an excellent catalytic performance towards the iodide electrolyte compared to the Pt standard counter electrode.^23^ It has been reported also that, Co_3_O_4_ has a good electro-catalytic activity towards reduction of I_3_^−^ ions into I^−^ ions.^24^ Furthermore, zinc substitution for Co in cobalt oxide(ii,iii) results in the spinel structure of Zn–Co–O with p-type conductivity. The hole transport layer of Zn–Co–O employed in DSSCs results in enhanced diffusion length and transport within a device.^25^ The DSSCs based spinel NiCo_2_O_4_ nanostructures reported high performance and are comparable to the Pt counter electrode.^26^ To address, the role of the local structure of the spinel-based cobalt materials in DSSCs performance still needs in-depth investigation.

In the present work, three-dimensionally arrays of spinel cobalt-based metal oxide thin films of MCo_2_O_4_ (M: Ni, Fe, Cu, Zn) were fabricated through hydrothermal deposition to use them as counter electrodes to obtain cost-effective DSSCs. Surface morphology and roughness of the prepared samples were investigated throughout SEM and AFM analysis. The XAFS technique is employed to examine the atomic coordination and interatomic distances by observing the local structure at Fe and Co K-edge. Also, the XANES spectra indicate the prepared samples have a spinel structure. Then, spinel cobalt-based metal oxide thin films were used in the fabrication of DSSCs as promising counter electrodes, and the photovoltaic performance of DSSCs with MCo_2_O_4_ counter electrodes were compared.

## Experimental procedures

The following materials are used to synthesis the spinel cobalt based oxide films: copper nitrate tetrahydrate (Cu(NO_3_)_2_·4H_2_O), iron monohydrate (Fe(NO_3_)_2_·H_2_O), nickel nitrate hexahydrate (Ni(NO_3_)_2_·6H_2_O), and zinc nitrate hexahydrate (Zn(NO_3_)_2_·6H_2_O), cobalt nitrate hexahydrate (Co(NO_3_)_2_·6H_2_O), hexamethylenetetramine (HMTA, (CH_4_)_6_N_4_), and indium tin oxide (ITO) coated glasses, all are purchased from Sigma Aldrich and used without any further purification.

**Fig. 1:**
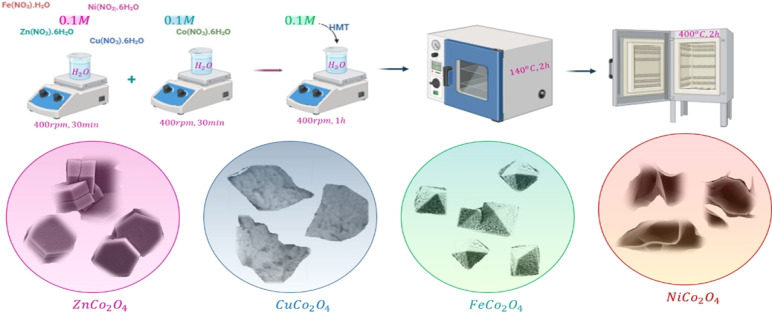
A schematic illustration depicting the deposition procedures of spinel cobalt-based samples.

The samples were grown hydrothermally using a solution processing route. The synthesis of spinel cobalt-based metal oxide thin films, MCo_2_O_4_, coated over a cleaned ITO substrate is shown in Fig. 1. The ITO substrate was subjected to cleaning in the acetone for 15 min followed by 15 min in the ethanol under sonication. The preparation procedure was performed by mixing 0.1 M of M(NO_3_)_2_·*x*H_2_O dissolved in 40 mL deionized water, and 0.1 M of Co(NO_3_)_2_·6H_2_O dissolved in 40 mL deionized (DI) water. The solution was then stirred at 400 rpm for 30 min. Then, the 0.1 M of (CH_2_)_6_N_4_ was added to the prepared aqueous solution. The obtained stock solutions were then transferred into a glass-sealed bottle containing a cleaned ITO substrate before being grown in an oven at 140 °C for two hours. Finally, the deposited spinel cobalt-based metal oxides of MCo_2_O_4_ films were rinsed with deionized water and annealed at 400 °C in a furnace for another two hours.

Scanning electron microscopy (SEM, Quanta FEG 450) was conducted to investigate the morphology and size distribution of the metal oxide samples. The surface roughness of the MCo_2_O_4_ thin films was carried out by using an atomic force microscope (AFM, SmartSPM™ 1000), FTIR (Bruker Vertex 80 and Hyperion 2000 microscope) used to investigate the vibrational modes and chemical bonds of metal oxides. The XAFS data were obtained *via* the BM-08 XAFS/XRF beamline at the Synchrotron-Light for Experimental Science and Applications in the Middle East (SESAME). The beamline was run in decay condition at an energy level of 2.5 GeV, with 300 mA as a maximum current. The X-ray absorption fine structure (XAFS) data were measured on the BM08-XAFS/XRF beamline of the SESAME operated at 2.5 GeV in “decay” mode with a maximum electron current of 300 mA.^27^ The XAFS spectra of the materials were acquired in transmission mode in the spectral range of Co K-edge (7709 eV) and Fe K-edge (7112 eV) at room temperature. The X-ray beam intensity was measured by ionization chambers filled with an optimal mixture of noble gases at a total pressure of 1.0 bar, and the XAFS data of the samples were acquired from the signals measured at ion chambers subsequently amplified by Stanford picoammeters and digitalized by a voltage to frequency converter, using a double-crystal Si (111) monochromator. The energy was calibrated at the Co K-edge (7709 eV) and Fe K-edge (7112 eV) of the Co and Fe standard metal foils, respectively. The sample was prepared in pallet form (13 mm diameter) by pressing a homogeneous mixture of calculated quantity of finely ground material and polyvinylpyrrolidone (PVP) powder. The amount of material in the pellet was calculated using XAFS mass software to give an absorption μt ∼1.5, just above the Co K-edge and Fe–K edge absorptions.

The DSSCs were prepared using TiO_2_ nanoparticle decorated ITO coated photoanodes. The electrodes were sintered at 450 °C in air ambient for 30 min to remove contaminations from the TiO_2_ nanoparticle surfaces. And then was immersed in N719 dye for a night. The photoanodes were washed with ethanol and dried using a heat gun. MCo_2_O_4_ coated ITO counter electrodes and dye-coated TiO_2_ photoanodes were sealed together and an electrolyte (iodide/tri-iodide in a nitrile solvent) was used to fill the space between two electrodes. The performances of DSSCs were measured under a class A solar simulator (ABET Technology Sun2000) with an AM 1.5 G filter and 100 mW cm^−2^ light intensity using a Keithley 2425 source meter.

## Results and discussion

**Fig. 2:**
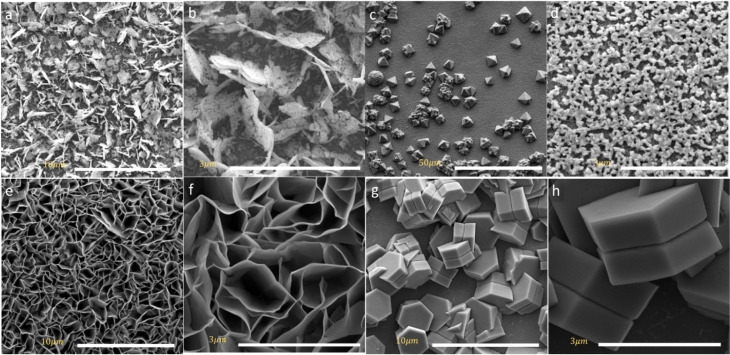
SEM images of the obtained (a and b) CuCo_2_O_4_ thin film (c and d) FeCo_2_O_4_ thin film (e and f) NiCo_2_O_4_ thin film and (g and h) ZnCo_2_O_4_ thin film.

The DSSCs device performance is directly influenced by the morphology of the counter electrode in particular redox/oxidation electrolyte at the interface. SEM images can give insight into crystal arrays and defective surfaces of spinel cobalt oxides. The surface morphologies of as-prepared MCo_2_O_4_ thin films are illustrated in Fig. 2. Interestingly, the morphology of the prepared samples can be correlated to the variations in source metal ions. Fig. 2(a) and (b) manifested uniform arrays of nano-leave structures that coincide with the CuCo_2_O_4_ thin film. Whereas Fig. 2(c) and (d) displays uniform arrays of octahedral-shaped structures observed in the FeCo_2_O_4_ thin film. The FeCo_2_O_4_ thin film is comprised of nano-blocks with average size of about 500 nm. The observed octahedron structure demonstrates high-pressure conditions controlled by the (111) plane, devoid of any preferential axis for selective growth.^28^Fig. 2(e) and (f) displays a hierarchical structure of NiCo_2_O_4_ nano-sheets observed with hollow arrays. Fig. 2(g) and (h) depicts a twin hexagonal configuration of nano-block ZnCo_2_O_4_ arrays observed with non-uniform distribution due to anisotropic growth along [100] and [001] directions.^29^ It is impressive to find that different Zn^2+^, Fe^2+^, Ni^2+^, and Cu^2+^ reacted species tend to form spinel structures in a cubic crystal phase. The intricate reaction pathways of these species during the hydrothermal process drive the formation of distinct morphologies of spinel metal oxides. Initially, the decomposed reacted species undergo a recrystallization process to form MCo_2_O_4_ with preferential growth on selective planes, depending on the nature of the ions. The synthesis of MCo_2_O_4_ is driven by the formation of M(OH)_2_ and Co(OH)_2_ before transforming into MCo_2_(OH)_6_ complex compound, as demonstrated by the NR Chodankar group.^30^ Alternatively, another reaction route suggests that the formation pathway is governed by the formation of Co_3_O_4_ and MO, which are then reduced to the MCo_2_O_4_ structure.^31^ The annealing process reduces the OH^−^ ions in the complex compound to the oxide form, resulting in the obtaining of bimetal oxides.

The HMT acts as an alkaline source that assists in the formation of M–Co precursor. During the hydrolysis of HMT, NH_3_ is released, making the solution alkaline. This process also produces OH^−^ ions in the hydrothermal synthesis, which react with Zn^2+^, Fe^2+^, Ni^2+^, Cu^2+^, and Co^2+^ ions to form spinel metal oxide. Furthermore, studies have found HMT useful for obtaining a crystalline spinel metal oxide structure. The alkaline environment facilitated by HMT is preferable for precipitating M^2+^ and Co^2+^ ions during hydrothermal deposition, accelerating the formation of the crystalline MCo_2_O_4_ structure.^32,33^

**Fig. 3:**
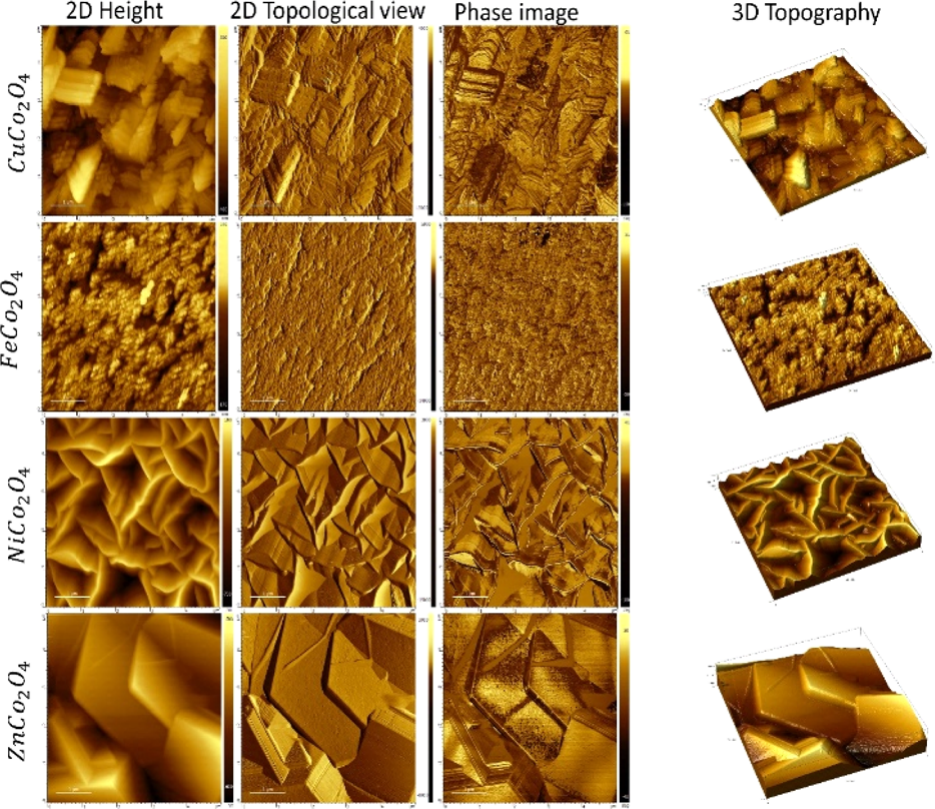
AFM images of as-deposited metal oxides of NiCo_2_O_4_, CuCo_2_O_4_, ZnCo_2_O_4_, NiCo_2_O_4_, and FeCo_2_O_4_ in 2D and 3D profile.

The surface area and roughness of the electrodes can provide significant insight into understanding the charge transfer mechanism and overall device performance. The AFM imaging was performed for the MCo_2_O_4_ thin films and illustrated in Fig. 3. The AFM images of the prepared CuCo_2_O_4_ thin film exhibit a high root mean square (RMS) roughness of 0.16 μm and a large surface area of 38.74 μm^2^. While the FeCo_2_O_4_ thin films show a polygon-shaped structure with an overall RMS of 0.03 μm and a surface area of 29.9 μm^2^. The phase image demonstrates a variation between – 50° and 20° indicating low phase separation at the grain boundaries. The NiCo_2_O_4_ nanosheets thin films depict a large surface area of 58.87 μm^2^ and RMS of 0.26 μm with an average thickness of nearly 20 nm, while a high roughness was observed for the hexagonal structure of ZnCo_2_O_4_ with an RMS of 0.35 μm and surface area of 34.9 μm^2^. The hexagonal plates of ZnCo_2_O_4_ are stacked in a non-uniform structural array.

In DSSCs, the device performance is highly dependent on the crystal quality of the counter electrode based a binary metal oxide films. The cubic spinel structure was confirmed for various metal oxide films from crystallographic X-ray diffraction plots in Fig. S1.† As seen, the diffracted XRD peaks of FeCo_2_O_4_ film match the (220), (311), (400), (422), and (440) crystal planes indexed to *Fd*3*m* space group (JCPDS card No. 04-0850). The crystal profile of NiCo_2_O_4_ nano-sheets assigned to (111), (220), (311), (222), (400), (331), and (511) crystal planes suggested *Fd*3*m* space group (JCPDS card No. 01-073-1702). For CuCo_2_O_4_ nano-leaves, the observed XRD profile in agreement with (111), (220), (311), (400), (422), and (511) crystallographic planes analogue to *Fd*3*m* space group (JCPDS card No. 23-1390). The ZnCo_2_O_4_ tends to form a crystal structure throughout hydrothermal synthesis similar to the above metal oxide films. The crystal diffraction profile of ZnCo_2_O_4_ resembles (220), (311), (222), (511), and (620) crystal planes for *Fd*3*m* (227) space group (JCDP card No. 00-001-1149).

The UV-vis-NIR spectral of the spinel metal oxide films plots in Fig. S2† and the corresponding extrapolated absorption band gap energies (*E*_g_) were determined according to Tauc plot. The NiCo_2_O_4_ nanosheets exhibit higher absorbance compared to the other spinel materials oxides followed by crystal CuCo_2_O_4_ nanoleaves. The straight-line interception yields two band gap energies of 1.98 and 2.50 eV for ZnCo_2_O_4_, while the obtained band gap energies of 2.00 and 3.35 eV for NiCo_2_O_4_ in agreement with the reported values of 2.00 and 3.30 eV.^34^ The valence band in NiCo_2_O_4_ was constructed from O 2p orbital and the conduction band from 3d orbitals of Ni and Co. And has an electron configuration of tetrahedral high spin Co^2+^ (eg^4^ t_2_g^3^), octahedral low spin Co^3+^ (t_2_g^6^), and Ni^3+^ (t_2_g^6^eg^1^). Therefore, the electron can be excited from Co-3d-t_2g_ to the partially filled Co 3d-eg orbital. Consequently, the presence of two band gap energies can be assigned to high spin and low spin of Co^3+^ in the spinel structure.^34^ The spinel CuCo_2_O_4_ band gap energies was 1.88 eV and 3.0 eV and FeCo_2_O_4_ yields 2.18 eV and 2.82 eV. The determined values are close to those reported in the literature.^35,36^

**Fig. 4:**
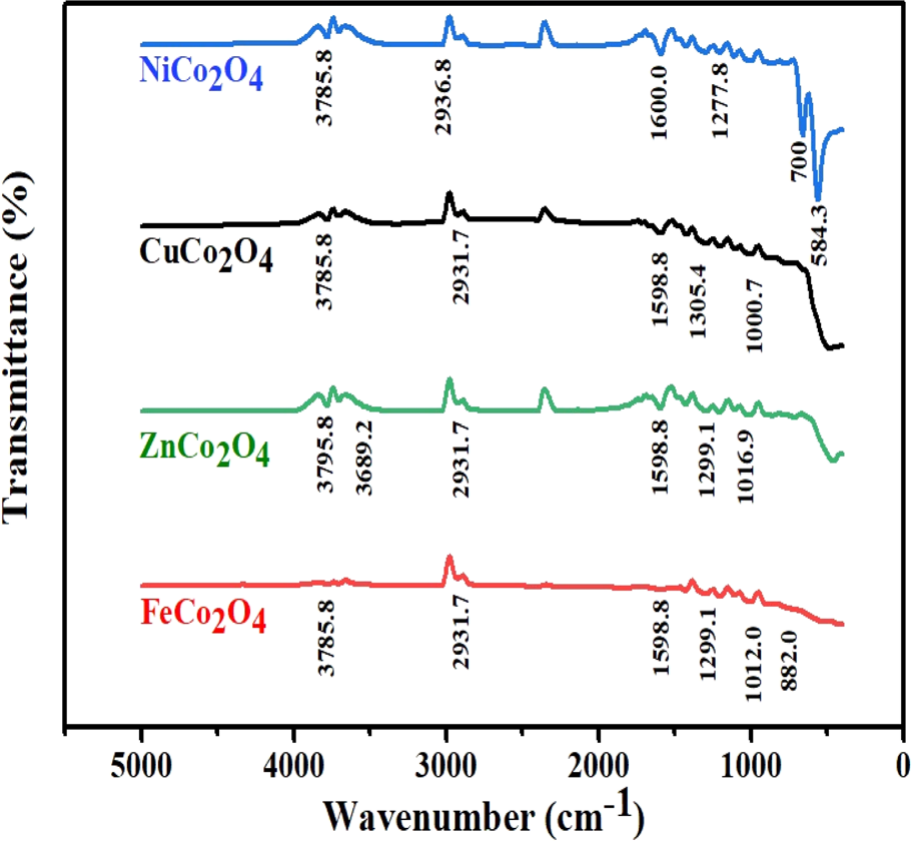
FTIR profile of the prepared binary metal oxide films MCo_2_O_4_.

The solution processed DSSCs device *via* hydrothermal usually contains –OH functional group. To verify the presence of the –OH group and other functional groups that could influence the device performance, the FTIR profile illustrated in Fig. 4 corresponds to the prepared MCo_2_O_4_ films. The vibration modes that match the M–O group can be seen at 584.3 cm^−1^ in various sample profiles. The M–O vibration modes at 584.3 cm^−1^ and 700 cm^−1^ may be associated with octahedral and tetrahedral sites.^37^ Furthermore, stretching modes located at approximately 882 cm^−1^ and 1000 cm^−1^ are assigned to the C–O group. Furthermore, the weak vibrational mode at 1277.8 cm^−1^ and 1299.1 cm^−1^ is linked to the NO_3_^−^ group.^38^ Additional stretching characteristics identified at 1598.8 cm^−1^ and 1600 cm^−1^ were assigned to COO and C=O molecules.^39^ The stretching modes at 2931.7 cm^−1^, 2936.8 cm^−1^, 3785.8 cm^−1^, and 3795.8 cm^−1^ are attributed to the O–H functional group.^40,41^ A higher content of OH– molecules was noticed in the case of ZnCo_2_O_4_ associated with a prominent shift in the OH vibration mode.

**Fig. 5:**
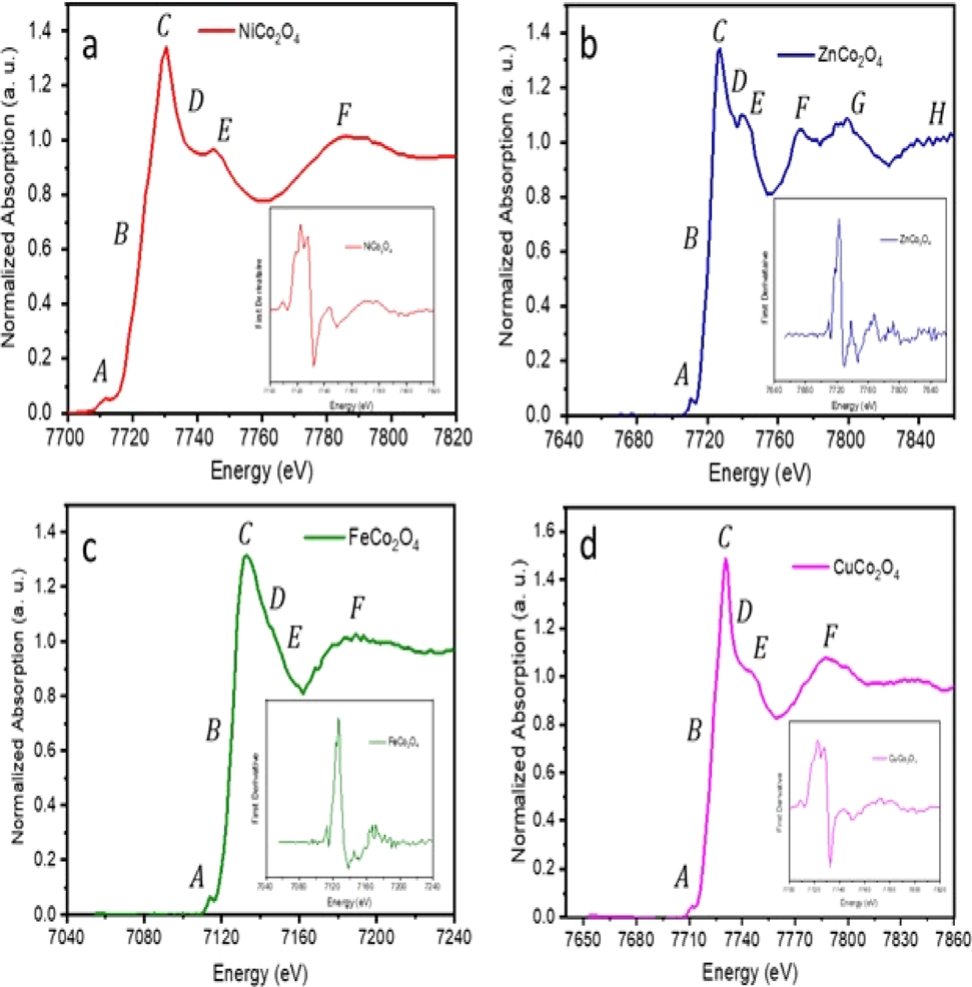
XANES spectra collected at Fe and Co K-edge of (a) NiCo_2_O_4_, (b) ZnCo_2_O_4_, and (c) CuCo_2_O_4_ collected at Co K-edge and (d) FeCo_2_O_4_ collected at Fe kedge; inset figures illustrate the corresponding first derivative of the absorption spectra.

The local structure of the MCo_2_O_4_ thin films could play an effective role in the DSSC device performance. The applied XAFS integrates to resolve the local structure of spinel cobalt oxide devices. The XANES spectra at Co absorption K-edge are presented in Fig. 5 of the prepared samples. Resolving the FeCo_2_O_4_ structure at the Co absorption K-edge is elusive and therefore XANES is applied at the Fe absorption K-edge. According to the XANES data, the Co absorption K-edge was (7722.53 eV) for NiCo_2_O_4_, (7724.02 eV) for CuCo_2_O_4_ and (7722.18 eV) for ZnCo_2_O_4_. The observed Co absorption K-edge aligns with the value reported in the literature at 7721.4 eV, while the Fe absorption K-edge was 7125.53 eV for FeCo_2_O_4_. The oxidation state of the cobalt cation is Co^3+^ and the observed XANES spectrum reveals the spinel structure of the prepared samples.^42,43^

The observed XANES spectrum reveals a low-intensity pre-edge peak at A, which can be assigned to 1s → 3d quadrupole transition in spinel cobalt oxide (Co_3_O_4_).^44^ A transition edge (shoulder region) was observed at B, along with the prominent white-line peak of high intensity at C. These transition edges at B and C can be attributed to resonance peaks.^45,46^ The white-line region can be ascribed to the 1s → np transition.^46^

Following that, minor peaks of low intensity were observed at D and E. The broadening of peak D, in particular, in FeCo_2_O_4_ can be attributed to the coordination atoms in the vicinity of the Co absorber atom. Moreover, two peaks at E and F indicate the presence of multiple scattering associated with the coordination of the medium-range structure surrounding the Co absorber atom.^47,48^ Furthermore, two peaks at G and H were observed in the XANES spectrum coinciding with the ZnCo_2_O_4_ sample, which can be attributed to the existence of multi-scattering effects.

To facilitate a comprehensive quantitative analysis of the local atomic structure of the MCo_2_O_4_ nanomaterials, the EXAFS fitting in R-space was conducted.^49^ The experimental EXAFS data in R-space in the range of 1.0 to 5.0 (Å) with Hanning window and k range of 3.0 Å^−1^ were analyzed to the best fit. The initial four high-ranking single scattering paths of Co–O and M–Co bond pairs were included in the fit. The passive electrons reduction factor *S*_0_^2^ and energy shift *E*_0_ were set similarly for all the paths in the fit, and the mean-square relative displacement *σ*^2^ and interatomic distance *R* were refined relatively to get the best fit result.

**Fig. 6:**
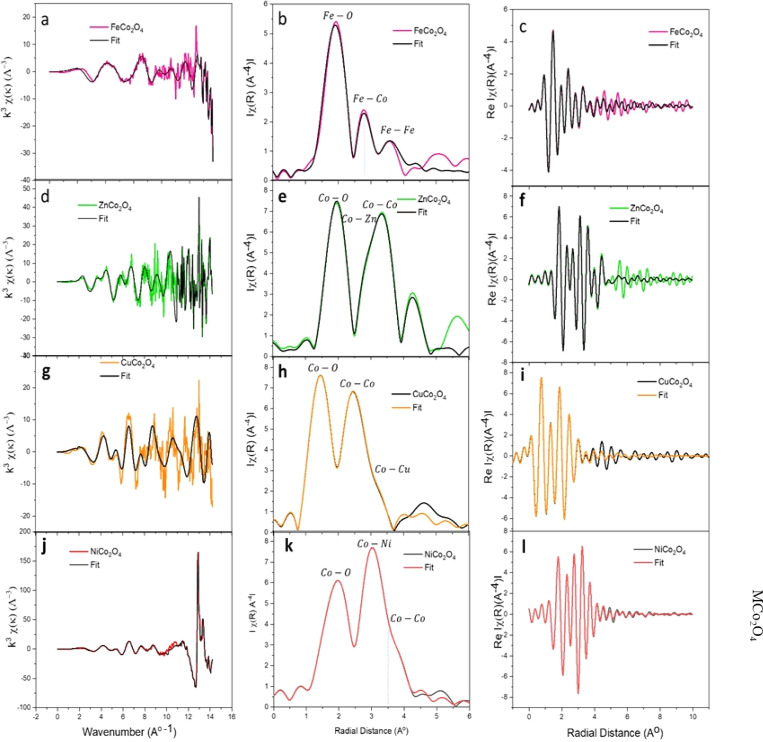
*k* ^3^-weighted EXAFS data with the best fit from Co and Fe K-edge absorption, FT, and the Re (FT) of the EXAFS data, respectively for (a–c) FeCo_2_O_4_, (d–f) ZnCo_2_O_4_, (g–i) Cu Co_2_O_4_ and (j–l) NiCo_2_O_4_ samples.

Fig. 6 plots *k*^3^-weighted EXAFS with the best fit, and the FT, respectively. The fit parameters with details are listed in Table 1. The FT analysis of spinel FeCo_2_O_4_ structure shows a strong peak around 1.500 Å assigned to the Fe–O_1_ (2.239 Å) coincides with T_d_ Fe^2+^ and Fe–O_2_ (1.963 Å) assigned to O_h_ Fe^3+^. The Fe–Co_1_ and Fe–Fe_1_ bonds are observed near the 3 Å which reveals the Co^2+^ cations are substituted by Fe^2+^ cations in the spinel Co_3_O_4_ structure (FeCo_2_O_4_). The Fe–Co^1^ can be resolved to O_h_ Fe^3+^ and T_d_ Co^2+^ sites. According to the reported data, the Fe, Cu, and Ni cations exhibit a tendency to occupy the octahedral sites (O_h_) and leave tetrahedral (T_d_) sites in spinel Co_3_O_4_.^50^ On the other side, the Co^2+^ ions in the Co_3_O_4_ compound are substituted by Zn^2+^ ions, which exhibit tetrahedral symmetry (T_d_), leading to the formation of ZnCo_2_O_4_.^46^ Interestingly, the ZnCo_2_O_4_ tends to form a spinel structure as the Zn^2+^ occupy the tetrahedral sites (T_d_) and leaving the Co^3+^ at octahedral sites (O_h_).^51^ The crystal ZnCo_2_O_4_ has a spinel structure (A^2+^Co_2_^3+^O_4_, where, A is tetrahedral and B is octahedral sites) closely similar to the Co_3_O_4_ structure.^51^ Indeed, the FT shows Co–O_1_ (1.966 Å) interaction, which is characteristic of O_h_ Co^3+^. Also, the Co–Zn_1_ bond suggested T_d_ Zn^2+^ and O_h_ Co^3+^ in the spinel structure of ZnCo_2_O_4_. The Co–O_1_ and Co–Co_1_ own octahedral symmetry. This finding shows a good agreement with the previous reports that confirmed the CoO_6_ structure has octahedral symmetry, whereas the CoO_4_ exhibits tetrahedral symmetry.^52^ Furthermore, the results imply that the Zn^2+^ cations have a tendency to occupy active sites characterized by a low oxidation state, whereas Co^3+^ cations are left in octahedral locations. It is clear that T_d_ Zn^2+^ and O_h_ Co^3+^ ions were presented in the crystalline structure of the ZnCo_2_O_4_ spinel structure.

**Table 1:** EXAFS fitting parameters, including coordination number (*N*), mean coordination shell radii (*R*), mean square relative displacements (MSRDs) or Debye–Waller factor (*σ*^2^), amplitude reduction factor (*S*_o_^2^), photoelectron energy (*E*_o_) and goodness of the fit (*R*_factor_) for the MCo_2_O_4_ binary metal oxides

Sample	Bond type	*N*	*R* (Å)	*σ* ^2^ (Å^2^)	*S*_o_^2^	*E*_o_ (eV)	*R*_factor_
CuCo_2_O_4_	Co–O_1_	2	1.917 ± 0.010	0.0023 ± 0.0044	1	0.530	0.1057
	Co–O_2_	2	2.058 ± 0.091	0.0147 ± 0.0423	1	0.530	0.1057
	Co–Co_1_	2	3.237 ± 0.150	0.0094 ± 0.0057	1	0.530	0.1057
	Co–Cu_1_	4	3.462 ± 0.067	0.0066 ± 0.0087	1	0.530	0.1057
NiCo_2_O_4_	Co–O_1_	2	1.912 ± 0.017	0.0009 ± 0.0020	1	0.679	0.0103
	Co–O_2_	4	1.940 ± 0.174	0.0238 ± 0.0072	1	0.679	0.0103
	Co–Ni_1_	4	2.835 ± 0.042	0.0037 ± 0.0088	1	0.679	0.0103
	Co–Co_1_	2	2.988 ± 0.078	−0.0015 ± 0.0099	1	0.679	0.0103
ZnCo_2_O_4_	Co–O_1_	4	1.966 ± 0.036	0.0045 ± 0.0023	1	0.250	0.0571
	Co–O_2_	6	3.808 ± 0.166	0.0045 ± 0.0268	1	0.250	0.0571
	Co–Zn_1_	4	3.197 ± 0.058	0.0057 ± 0.0038	1	0.250	0.0571
	Co–Co_1_	6	3.439 ± 0.234	0.0001 ± 0.0303	1	0.250	0.0571
FeCo_2_O_4_	Fe–O_1_	2	2.239 ± 0.173	0.0056 ± 0.027	1	0.527	0.0320
	Fe–O_2_	4	1.963 ± 0.105	0.0045 ± 0.0054	1	0.527	0.0320
	Fe–Co_1_	4	3.224 ± 0.252	0.0006 ± 0.0123	1	0.527	0.0320
	Fe–Fe_1_	2	3.409 ± 0.266	−0.0015 ± 0.012	1	0.527	0.0320

Moreover, the FT first peak at 1.900 Å interatomic distance in spinel CuCo_2_O_4_ structure corresponds to the Co–O_1_ (1.917 Å) of Oh Co^3+^ and Co–O_2_ (2.058 Å) of T_d_ Co^2+^. The results in accordance with the previous reports.^51,53^ The FT peak at 3.200 Å is correlated to Co–Co_1_ (3.237 Å) and Co–Cu1 (3.462 Å) bond interactions. It has been reported that the Co–Co interactions can be found in Oh and Td as Co^3+^–Co^3+^ (2.85 Å), Co^3+^–Co^2+^ (3.346 Å), and Co^2+^–Co^2+^(3.495 Å).^54^ Therefore, the Co–Co1 interactions in spinel CuCo_2_O_4_ assigned to O_h_ Co^3+^ and T_d_ Co^2+^. Moreover, the Co–Cu_1_ bridges the T_d_ Cu^2+^ and T_d_ Co^2+^.

The above-reported XANES (Fig. 5b) pre-edge of NiCo_2_O_4_ suggested the presence of T_d_ Co^2+^ symmetry.^55^ It can be preliminary concluded that, the NiCo_2_O_4_ is close to the spinel Co_3_O_4_ structure and the oxidation state for both is 8/3 as oxidation state corresponding to the presence of O_h_ Ni^3+^.^56^ The FT analysis of NiCo_2_O_4_ reveals a peak at the lowest interatomic distance of 1.94 Å assigned to Co–O_1_ of O_h_ (Co^3+^O_6_) and Co–O_2_ of T_d_ (Co^2+^O_4_).^57^ The next FT peak at 2.9 Å corresponds to Co–Co_1_ (2.988 Å) and 4Co–Ni_1_ (2.835 Å) interactions. It's evident the Co–Co_1_ is characteristic of Oh Co^3+^ and the Co–Ni1 coincides characteristic of O_h_ Co^3+^ and the Co–Ni_1_ coincides with O_h_ Co^3+^ and O_h_ Ni^3+^. In general, the Co–O interatomic distances at T_d_ or O_h_ sites are shorter than M–O distances which is correlated with a small atomic radius of Co^3+^ in comparison with M^2+^. It is obvious that the Co–O_1_ and Co–O_2_ in ZnCo_2_O_4_ are longer compared to other spinel structures. It is worth noting that, the interatomic distances of M–Co introduced in Table 1 are observed as Co–Cu > Co–Fe > Zn–Co > Co–Ni.

**Fig. 7:**
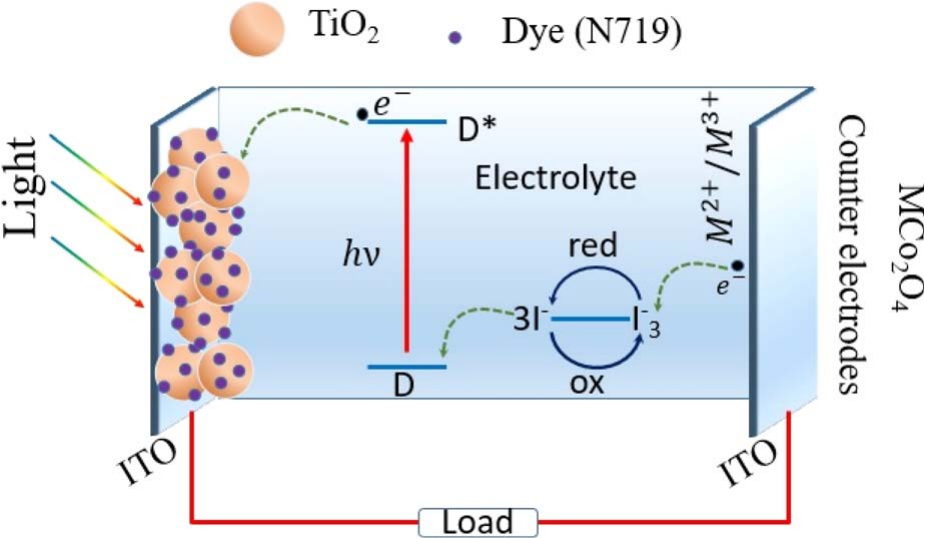
Schematic diagram of MCo_2_O_4_ counter-electrode-based DSSC.

The working mechanism of DSSCs in this study, as illustrated in Fig. 7, involves several key components and processes. The TiO_2_ nanoparticle-decorated ITO-coated photoanode serves as the light-harvesting layer. When light is absorbed by the sensitizer dye (here N719), it excites electrons from the dye into the conduction band of the TiO_2_. These photo-generated electrons then move through the TiO_2_ nanoparticles and are collected at the ITO substrate, flowing into the external circuit to generate electric current. The oxidized dye molecules are regenerated by electrons from the iodide (I^−^) in the electrolyte, which is subsequently oxidized to tri-iodide (I_3_^−^). The tri-iodide ions diffuse towards the spinel cobalt-based metal oxide MCo_2_O_4_ thin film counter electrodes, where they are reduced back to iodide ions, completing the circuit. This process is facilitated by the high electrocatalytic activity of the MCo_2_O_4_ counter electrodes, which efficiently catalyzes the reduction of tri-iodide. The high surface area and conductivity of the MCo_2_O_4_ thin films can play an important role in efficient electron transfer and low charge transfer resistance, contributing to the high performance of the DSSCs. Additionally, the chemical and thermal stability of MCo_2_O_4_ provides durability and long-term performance stability, making it an excellent choice for counter electrodes in high-performance DSSCs.

**Fig. 8:**
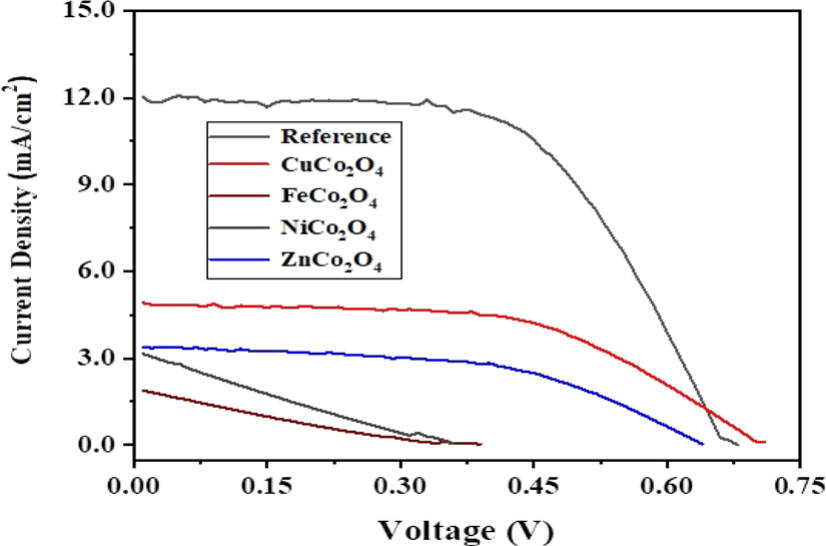
Current density–voltage plots of DSSCs formed using Pt and MnCo_2_O_4_-based counter electrodes.

Fig. 8 shows current density–voltage (*J*–*V*) plots of the DSSCs fabricated using sensitized MCo_2_O_4_ based counter electrodes in comparison with Pt-based one as reference solar cell under illumination of 100 mW cm^−2^. Table 2 summarizes the obtained results and highlights significant differences among the examined thin films. As presented in Table 2, the DSSC with a counter electrode based on CuCo_2_O_4_ nano-leave gives the best photovoltaic response with a *J*_max_ of 4.23 mA cm^−2^ and a *V*_max_ of 550 mV which results in 1.9% power conversion efficiency. It is also seen that the open circuit voltage (*V*_OC_) value of this device (705.8 mV) superior to the one for conventional DSSC with Pt electrode (664.1 mV). The second-best solar cell efficiency is reported as 1.13% for the DSSC obtained using ZnCo_2_O_4_ hexagonal-like structures. The *V*_OC_ value of this device with 642.0 mV is very close to the cell with Pt electrode. The other two solar cells obtained from NiCo_2_O_4_ nanosheets and FeCo_2_O_4_ diamond-like structures have lower PCE values of 0.27 and 0.15%, respectively. The lower short-circuit current density (*J*_SC_) values observed in our study for DSSCs utilizing alternative counter electrodes, such as CuCo_2_O_4_, FeCo_2_O_4_, NiCo_2_O_4_, and ZnCo_2_O_4_, compared to conventional Pt-based DSSCs. It suggests potential limitations in transport mechanisms associated with these materials. Factors such as differences in catalytic activity, electronic structure, and surface morphology may contribute to the reduced efficiency of photogenerated charge carriers and subsequent lower *J*_SC_ values. Similar studies have been performed for various metal oxides including binary metal oxides.^58–60^ For instance, Kaya *et al.* hydrothermally sensitized CuCrO_2_ delofossite oxide nanoparticles to use them as photocathode in the fabrication of high efficient tandem p–n photoelectrochemical cells using CuCrO_2_ delafossite semiconductors as photocathodes coupling with traditional n-type TiO_2_ based photoanodes.^59^ They reported the efficiency of solar cells between 1.67 and 2.33% for various annealing temperatures.

**Table 2:** Some photovoltaic parameters of the DSSCs fabricated using Pt and MCo_2_O_4_-based counter electrodes

DSSCs	*J*_max_ mA cm^−2^	*V*_max_ mV	*J*_sc_ mA cm^−2^	*V*_oc_ mV	FF	PCE %
CuCo_2_O_4_	4.23	550	4.80	705.8	56.1	1.90
FeCo_2_O_4_	0.99	150	1.96	401.8	18.8	0.15
NiCo_2_O_4_	1.67	160	3.25	364.9	22.6	0.27
ZnCo_2_O_4_	2.83	400	3.41	642.0	51.7	1.13
Pt	10.81	440	12.1	664.1	59.2	4.76

Considering the structural properties examined in this study across various spinel metal oxides, the samples consistently demonstrated high crystallinity, primarily in the cubic phase. Notably, the identification of active structural planes in these samples indicates a correlation between electrochemical activity and charge injection with these specific planes. For instance, the (311) plane was prominent in the ZnCo_2_O_4_ structure, the (220) plane in CuCo_2_O_4_, both (311) and (220) planes in NiCo_2_O_4_, and the (220) plane in FeCo_2_O_4_. Furthermore, distinctive characteristics and morphologies were observed in the hexagonal structure of ZnCo_2_O_4_ and the nanosheet morphology of CuCo_2_O_4_, which are thought to enhance the photocurrent and efficiency of DSSCs.

## Conclusions

The spinel cobalt-based metal oxides of MCo_2_O_4_ (M: Ni, Cu, Fe, or Zn), were synthesized through the hydrothermal growth technique on ITO substrate to use them as counter electrodes in the fabrication of Pt free DSSCs. The oxides displayed a uniform and homogeneous surface morphology. Interestingly, NiCo_2_O_4_ exhibits nanosheets, Fe Co_2_O_4_ displays diamond shapes, CuCo_2_O_4_ shows nanoleaves, and ZnCo_2_O_4_ presents a hexagonal shape. These morphologies exhibit unique structural features characterized by a significantly large surface area and the existence of surface defect sites to enhance chemical reactivity. To probe the local structure of the prepared samples, EXAFS data was carried out at Fe and Co K-edge. The absorption Co K-edge was close to the 7721.4 eV value reported in the literature. The Fe Kedge was 7125.53 eV for FeCo_2_O_4_. The pre-edge peak in XANES of the prepared samples suggested T_d_ Co^2+^. The shape of the XANES spectrum reveals the spinel structure of the obtained samples. The FT reveals a Co–O_1_ coincides with O_h_ Co^3+^ and Co–O_2_ is characteristic for T_d_ Co^2+^. On the contrary, Fe–O_1_ reveals T_d_ Fe^2+^ and Fe–O_2_ associated with O_h_ Fe^3+^. The interatomic distances evaluated from FT analysis of M–Co were observed as Co–Cu > Co–Fe > Zn–Co > Co–Ni. In addition, it is reported that these spinels cobalt-based binary metal oxides, especially CuCo_2_O_4_ nanoleaves and ZnCo_2_O_4_ hexagonal-like structures are promising candidates for the low cost and high efficient DSSCs with 1.9 and 1.13% power conversion efficiency values, respectively.

## Data availability

The data that support the findings of this study are available from the corresponding author, Bashar Aljawrneh, upon reasonable request. Researchers interested in accessing the data should contact Bashar Aljawrneh at b.aljawarneh@zuj.edu.jo.

## Author contributions

Abdelelah Alshanableh: conceptualization, investigation, methodology, data curation, writing – original draft, writing – review & editing. Yusuf Selim Ocak: conceptualization, investigation, methodology, data curation, writing – original draft, writing – review & editing. Bashar Aljawrneh: conceptualization, investigation, methodology, data curation, writing – original draft, writing – review & editing. Borhan Aldeen Albiss: supervision, investigation, review & editing, funding acquisition. Khaled Shawakfeh: supervision, investigation, review & editing, funding acquisition. Latif U. Khane: investigation, methodology, data curation, writing – original draft. Messaoud Harfouchee: investigation, methodology, data curation, writing – original draft. Saja Alrousan: investigation, methodology, data curation.

## Conflicts of interest

There are no conflicts to declare.

## Supplementary Material

RA-014-D4RA03462G-s001
