# Correction: Spinel cobalt-based binary metal oxides as emerging materials for energy harvesting devices: synthesis, characterization and synchrotron radiation-enabled investigation

**DOI:** 10.1039/d4ra90082k

**Published:** 2024-08-09

**Authors:** Abdelelah Alshanableh, Yusuf Selim Ocak, Bashar Aljawrneh, Borhan Aldeen Albiss, Khaled Shawakfeh, Latif U. Khan, Messaoud Harfouche, Saja Alrousan

**Affiliations:** a Nanotechnology Institute, Jordan University of Science & Technology PO Box 3030 Irbid 22110 Jordan; b Department of Physics and Engineering Physics, Morgan State University Baltimore Maryland 21234 USA; c Department of Physics, Al-Zaytoonah University of Jordan PO Box 130 Amman 11733 Jordan B.Aljawarneh@zuj.edu.jo; d Department of Chemistry, Jordan University of Science & Technology PO Box 3030 Irbid 22110 Jordan; e Synchrotron-Light for Experimental Science and Applications in the Middle East (SESAME) PO Box 7 Allan 19252 Jordan

## Abstract

Correction for ‘Spinel cobalt-based binary metal oxides as emerging materials for energy harvesting devices: synthesis, characterization and synchrotron radiation-enabled investigation’ by Abdelelah Alshanableh *et al.*, *RSC Adv.*, 2024, **14**, 21180–21189, https://doi.org/10.1039/D4RA03462G.

The authors regret that the names of three of the authors (Khaled Shawakfeh, Latif U. Khan and Messaoud Harfouche) were shown incorrectly in the original article. The corrected author list is as shown above.

The Royal Society of Chemistry apologises for these errors and any consequent inconvenience to authors and readers.

