# Supplementary material for: Spinel cobalt-based binary metal oxides as emerging materials for energy harvesting devices: synthesis, characterization and synchrotron radiation-enabled investigation
Source: RSC Adv. 2024 Jul 4;14(29):21180–9. doi: 10.1039/d4ra03462g (PMC11223668; doi:10.1039/d4ra03462g)

Electronic Supplementary Material (ESI) for RSC Advances.
This journal is © The Royal Society of Chemistry 2024

Supporting Information

Spinel Cobalt-Based Binary Metal Oxides as Emerging Materials for Energy Harvesting Devices: Synthesis, Characterization and Synchrotron Radiation-Enabled Investigation

Abdelelah Alshanableh,[a] Yusuf Selim Ocak,[a,b] Bashar Aljawrneh,*[,c] Borhan Aldeen Albiss,[a] Khaled Shawakfehc,[d] Latif U. Khane,[e] Messaoud Harfouchee,[e] and Saja Alrousan[a]

[a]*Nanotechnology Institute, Jordan University of Science & Technology, P.O. Box 3030, Irbid-22110, Jordan.*

[b]*Department of Physics, Al-Zaytoonah University of Jordan, P.O. Box 130, Amman-11733, Jordan.*

[c]*Department of Chemistry, Jordan University of Science & Technology, P.O. Box 3030, Irbid-22110, Jordan.*

[d]*Smart-Lab, Dicle University, Diyarbakir, 21020, Turkiye*

[e]*Synchrotron-light for Experimental Science and Applications in the Middle East (SESAME) P.O. Box 7, Allan 19252, Jordan*

Figure S1: XRD patterns of various spinel cobalt-based metal oxide films

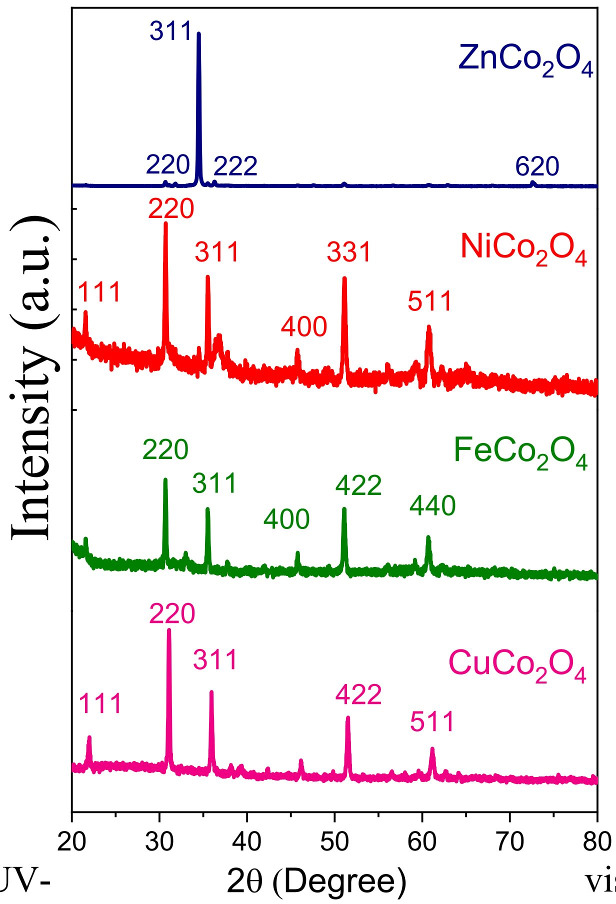

Figure S2: The UV-vis-NIR absorption

spectrum of spinel cobalt based metal oxide films and the corresponding band gap energies

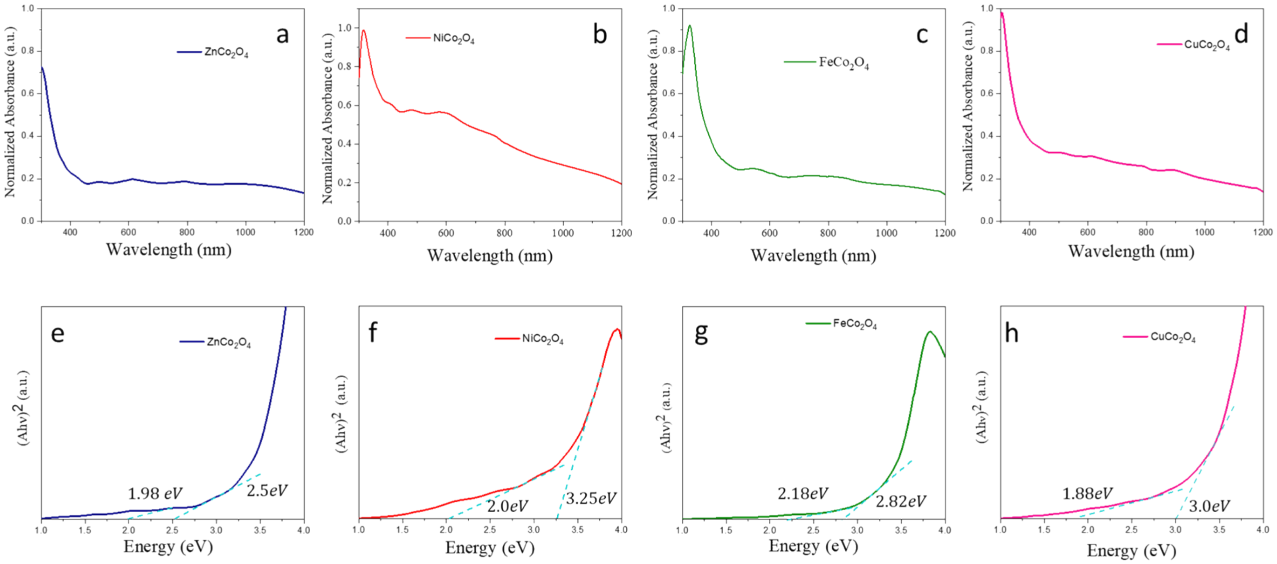

Supplement: RA-014-D4RA03462G-s001 [file RA-014-D4RA03462G-s001.pdf]
